# The Effectiveness of Hydrodissection with 5% Dextrose for Persistent and Recurrent Carpal Tunnel Syndrome: A Retrospective Study

**DOI:** 10.3390/jcm11133705

**Published:** 2022-06-27

**Authors:** Ta-Chung Chao, Kenneth Dean Reeves, King Hei Stanley Lam, Tsung-Ying Li, Yung-Tsan Wu

**Affiliations:** 1 Department of Physical Medicine and Rehabilitation, Tri-Service General Hospital, School of Medicine, National Defense Medical Center, Taipei 114, Taiwan; ericapple3000@yahoo.com.tw (T.-C.C.); doc31141@gmail.com (T.-Y.L.); 2 Private Practice PM&R and Pain Management, Roeland Park, KS 662, USA; deanreevesmd@gmail.com; 3 The Hong Kong Institute of Musculoskeletal Medicine, Hong Kong; drlamkh@gmail.com; 4 Department of Family Medicine, The Chinese University of Hong Kong, Hong Kong; 5 Department of Family Medicine, The University of Hong Kong, Hong Kong; 6 Center for Regional Anesthesia and Pain Medicine, Wan Fang Hospital, Taipei Medical University, Taipei 116, Taiwan; 7 Integrated Pain Management Center, Tri-Service General Hospital, School of Medicine, National Defense Medical Center, Taipei 114, Taiwan; 8 Department of Research and Development, School of Medicine, National Defense Medical Center, Taipei 114, Taiwan

**Keywords:** dextrose, carpal tunnel syndrome, hydrodissection, surgery

## Abstract

Patients with failure of primary surgery for carpal tunnel syndrome (CTS) present a frustrating clinical problem because there are no relevant treatment guidelines, and the effect of current conservative management or revision surgery is unsatisfactory. Hydrodissection with 5% dextrose is emerging as an effective treatment for primary CTS and may be an effective alternative treatment method for persistent or recurrent post-surgical CTS. We retrospectively investigated the long-term effectiveness of hydrodissection with 5% dextrose for persistent or recurrent CTS. Thirty-six of forty consecutively-treated patients with either persistent or recurrent symptoms of CTS after surgery, who were treated with ultrasound-guided hydrodissection of the median nerve using 10 mL of 5% dextrose, were available to provide outcome data by a structured phone interview at least six months after treatment completion. Symptom relief ≥ 50% represented an effective outcome, while symptom relief < 50% was rated as a poor outcome. Nearly 2/3 (61.1%) of patients reported an effective outcome after a mean of 3.1 injections, with a post-injection follow-up mean of 33 (6–67) months. A non-significant trend toward a more frequently-effective outcome was observed in those with recurrent versus persistent symptoms following CTS (76.9% vs. 52.2%, *p* = 0.165). However, a significantly higher percentage of those with recurrent symptoms reported an excellent outcome, defined as a greater than 70% improvement (8/13 [61.6%] vs. 3/23 [13%], *p* = 0.006). The percentage of patients achieving an effective outcome was not significantly different between <2, 2–4, and >4 years of post-treatment follow-up (36.4% vs. 77.8% vs. 57.1%; *p* = 0.077). Hydrodissection with 5% dextrose may result in a clinically important and durable benefit in those experiencing persistent or recurrent CTS after surgery.

## 1. Introduction

Carpal tunnel syndrome (CTS), which is the most common (accounting for 90% of all cases) peripheral entrapment neuropathy, is considered to involve excess pressure on the median nerve (MN) as it passes through the carpal tunnel [1]. Increased intracarpal pressure contributes to the interruption of nerve microcirculation, ischemia, impaired nerve conduction, and decreased MN dynamics with adhesion [2,3]. The worldwide CTS incidence is 3–4%, with typical symptoms and signs including numbness, tingling, pain, burning sensation, or nocturnal paresthesia. In addition, weakness with thenar muscle atrophy might occur in subsequent stages or severe cases [4,5].

The management of CTS includes conservative or surgical treatments depending on symptom severity [6,7]. Conservative treatments (splint use, corticosteroid injection, medications, physical therapy, etc.) are recommended for mild-to-moderate CTS. However, these treatments have limited clinical benefit, and a recent systematic review revealed that 57% to 66% of patients underwent surgery after receiving conservative treatments for 1–3 years [1]. Although surgery is considered to have better mid-and long-term effects than conservative management, patients with failure of primary surgery for CTS remain a frustrating clinical problem. They can be considered in three categories: persistent, recurrent, or new, and the vast majority are persistent or recurrent [8]. There are no satisfactory evidence-based non-surgical treatment guidelines for CTS patients who have failed primary surgery [8,9]. Many patients undergo revision surgery, followed by persistent symptoms in 41% to 90% after revision. Only 40% to 47% of patients have satisfactory improvement after revision surgery [10,11,12]. Identification and confirmation of an effective non-surgical treatment to improve the success rate for patients with failure of primary surgery is a critical need area.

In 2017, Wu et al. [13] reported that ultrasound-guided hydrodissection with 5 mL 5% dextrose (D5 hydrodissection; D5HD) was successful in the treatment of mild-to-moderate CTS and outperformed corticosteroid injection [14]. D5HD is emerging as an effective primary conservative treatment for CTS based on multiple favorable clinical trials [13,14,15,16,17,18,19,20] and favorable meta-analyses [21,22,23]. In a recent retrospective study of the effects of D5HD, we included 15 patients treated for persistent or recurrent symptoms after surgery, with an “excellent” or “good” outcome in 12 of 15 at a 15.3-month post-injection follow-up [17]. The goal of our study was to confirm the findings in a larger population of patients with recurrent or persistent CTS symptoms, followed by a more extended period to confirm the durability of the benefit.

## 2. Materials and Methods

### 2.1. Study Design

This retrospective study was approved on 26 April 2021, after a review by the institutional review board of the Tri-Service General Hospital, School of Medicine, National Defense Medical Center, Taipei, Taiwan (TSGHIRB No. B202105014), and the requirement for written/signed agreement of informed consent was waived. All patients treated between November 2016 and November 2021 for persistent or recurrent CTS after primary surgery by D5HD were identified through chart review and contacted by phone. All patients had received treatment from the same physician at a single medical center. Baseline demographic data, symptom duration prior to D5HD and ultrasonographic cross-sectional area (CSA) of MN were reviewed.

### 2.2. Inclusion and Exclusion Criteria

The clinical diagnosis of persistent or recurrent CTS required the presence of a weak and clumsy hand with paresthesias/dysesthesias aggravated by repetitive wrist use or during sleep, and one of the following: (1) Numbness in the MN innervated region, (2) Wasting of the thenar muscle with weakness, or (3) Phalen’s test ± Tinel’s sign positive. Patients 20 to 80 years of age with a pain intensity of ≥4/10 on the visual analog scale (VAS) prior to treatment, with either persistent CTS symptoms, ≥one month after carpal tunnel release, or recurrent symptoms after a symptom-free postoperative interval of 6 months or more [12,19], were candidates for inclusion. We excluded patients who could not be contacted by phone, refused to participate, or had undergone revision surgery for CTS.

### 2.3. Primary Measures

Structured telephone interviews began in April 2022, conducted by the same research assistant as in previous retrospective studies [17,24]. The investigator asked all patients about the percentage of symptom relief of their affected hand(s) (digital pain severity, paresthesia, or dysesthesia associated with CTS) post-injection compared to pre-injection using VAS as in previous studies [17,24]. We categorized the patient-reported outcomes as follows: (1) Symptom relief ≥ 70% as an excellent outcome, (2) Symptom relief ≥ 50% but <70% as a good outcome, (3) Symptom relief ≥ 30% but <50% as slight improvement, (4) Symptom relief < 30% as no change, and (5) Symptom worse than pre-injection as a worse outcome. Only excellent or good outcomes were considered effective outcomes; otherwise, patients were rated as having poor outcomes [17,24]. For patients with bilateral CTS, only the most symptomatic side was recorded for the analysis. We also asked the patients if they underwent any other treatments because of unremitting symptoms of CTS after D5HD.

### 2.4. Ultrasound-Guided Hydrodissection with 5% Dextrose

A single physician with 7 years of experience using an ultrasound-guided perineural injection performed the entire procedure using a 10–18-MHZ linear array transducer (MyLab™25Gold, Esaote, Genova, Italy). A 25-gauge, 2-inch needle was used without local anesthetic. A total of 10 mL of 5% dextrose was used for hydrodissection. Summarily, by way of an in-plane ulnar approach at the proximal inlet of the carpal tunnel (scaphoid-pisiform level), the MN was dissected from the subsynovial connective tissue (SSCT) through a short-axis view with 4 mL 5% dextrose. Next, 4 mL 5% dextrose was injected via short-axis to hydrodissect the MN apart from the flexor retinaculum (FR). Then, the remaining 2 mL of 5% dextrose was proximally to distally hydrodissected longitudinally to further separate the MN from the FR [17]. Examination of the MN course in each case confirmed that the nerve had been freed from the fascia throughout the canal, and had assumed a rounded/oval appearance, instead of an elliptical appearance, as the endpoint [25,26,27].

### 2.5. Statistical Analysis

Data were statistically analyzed using SPSS statistics version 22 (IBM, Armonk, NY, USA). The Mann–Whitney *U* test or Fisher’s Exact Test were used to analyze categorical or continuous demographic data, respectively, between groups. Comparisons between different follow-up periods were analyzed using the Kruskal–Wallis test or a within-group *T*-test. Statistical significance was considered as a *p*-value < 0.05.

## 3. Results

Forty consecutively treated patients met the inclusion criteria. Thirty-six were able to be contacted and all thirty-six were willing to be interviewed about follow-up status. Data from 15 of 36 participants had already been gathered in a previous retrospective study of D5HD that included some participants who had failed CTS, although their results were confirmed/enhanced by a long-term follow-up [17]. There were no significant between-group differences in demographic measures, or in the number of participants who received additional treatment (Table 1). Treatment by D5HD began long enough post-surgery that spontaneous remission was unlikely (21.2 ± 4.2 months; data not shown). Participants were followed up a mean of 33 ± 2.8 months after their last treatment with D5HD and none had received revision surgery. The mean age was 59.2 ± 1.6 years, and the mean symptom duration was 15.1 ± 2.4 months. The mean number of injection sessions was 3.1 ± 0.3, with a 1- to 4-week interval between each injection. CTS symptoms were categorized as persistent or recurrent in 23/36 (63.9%) and 13/36 (36.1%), respectively. Twenty-two of the thirty-six patients (61.1%) reported an effective outcome and fourteen (38.9%) reported a poor outcome. No patients reported worsening due to treatment. Eleven patients (five in the effective and six in the poor outcome group) received other conservative management, e.g., acupuncture, rehabilitation, and medication (Table 1).

**Figure 1:**
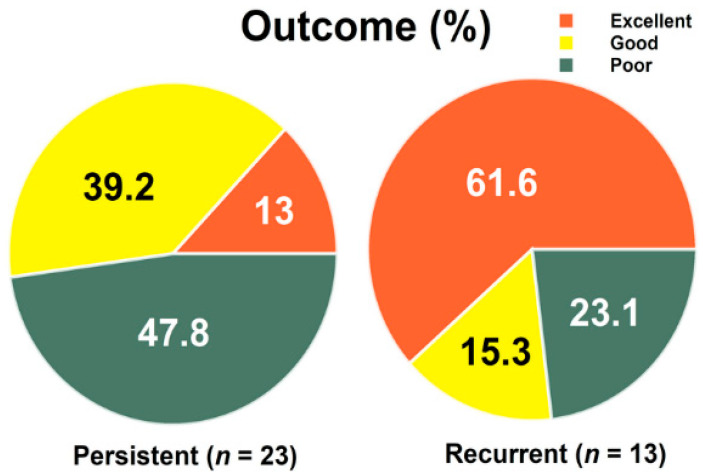
The percentage of outcomes among persistent and recurrent carpal tunnel syndrome. Recurrent vs. persistent (Excellent outcome; 8/13 [61.6%] vs. 3/23 [13%], *p* = 0.006), two-way Fisher’s Exact test.

A non-significant trend toward a more frequently-effective (excellent or good) outcome was observed in those with recurrent versus persistent symptoms following CTS (76.9% vs. 52.2%, *p* = 0.165) (Table 2). The percentage of patients achieving an effective outcome was not significantly different between <2, 2–4, and >4 years of post-treatment follow-up (36.4% vs. 77.8% vs. 57.1%, *p* = 0.077) (Table 3). A total of 13%, 39.2% and 47.8% of patients in the persistent group reported excellent, good and either minimal or poor outcomes, respectively, and 61.6%, 15.3%, and 23.1% in the recurrent group did so. This between-group distribution was significantly different (*p* = 0.01), and specifically those with recurrent symptoms were more likely to achieve an excellent outcome (≥70% improvement; 8/13 [61.6%] vs. 3/23 [13%], *p* = 0.006) (Figure 1).

## 4. Discussion

This first retrospective study, exclusively of those with persistent or recurrent carpal tunnel symptoms after CTS surgery, showed an effective outcome in 61% of patients with a mean of 3.1 injections of D5HD, and a durable outcome, with a mean post-injection follow-up of 33 months. This suggests that D5HD may be an effective treatment for CTS patients with failure of primary surgery, and points to the need for prospective and potentially controlled studies.

Although most CTS patients are satisfied with surgery, there is a certain percentage (3–20%) of individuals who fail to achieve or maintain adequate benefit from surgery [8]. Persistent CTS is the most common presentation, accounting for about 43% of revision surgery patients [11]. The main etiology is an incomplete release of FR (about 50–58%), and other causes include a secondary site of compression, an irreversible compressive neuropathy, or an inaccurate preoperative diagnosis [28]. Recurrent symptoms are the second most common presentation (about 4–57%) [11,29], and the main etiology is perineurial adhesions (about 88%) with less frequent causes including reconstitution of the FR or development of a secondary site of compression. New symptom onset is the least frequent presentation and may be caused by iatrogenic nerve injury [28]. Irrespective of presentation, there is a paucity of high-level research exploring management for CTS surgery failure, and therapeutic outcomes are disappointing. Conservative treatments, including splinting, corticosteroid injections, activity modification, and exercises designed to promote tendon and nerve gliding should be utilized. Revision surgery may be necessary if symptoms fail to improve after conservative treatment. Revision surgery includes revision neuroplasty, neurolysis, nerve reconstruction, or local soft-tissue flap coverage, with favorable outcomes less consistent compared to primary surgery [30,31,32].

The mechanism of D5HD for CTS can be divided into mechanical and pharmacological effects. The mechanical effect, called hydrodissection, which can detach the nerve by a non-specific effect of fluid-under-force, may progressively lessen adhesions, increase blood flow, and remobilize the nerve [19,25,27,33,34,35]. Compared with the logical mechanical benefits of hydrodissection, the pharmacological effects of dextrose on nerves are not clear. Several researchers have proposed that elevated glucose may stabilize neural activity, regulate glucose metabolism or decrease neurogenic inflammation to lessen neuropathic pain via multifactorial mechanisms. When the nerve is in a hypoglycaemic environment, histopathological changes result in the peripheral nerves, along with the activation of nociceptive C-fibers, with increased noxious signal transduction [36,37]. The excessive activation of nociceptive C-fiber nerves will quickly return to normal after adding glucose [36]. Moreover, the elevated extracellular glucose concentration could hyperpolarize C-fibers to stabilize their activation [38]. Adenosine monophosphate protein kinase (AMPK) is a key enzyme regulating cell metabolism, and decreased AMPK activity is associated with neuropathic pain and vice versa [39,40]. AMPK activity may decrease with hypoglycemia and return to normal with elevated glucose, which may be associated with pain reduction [40]. Elevated glucose may be neuroprotective in other ways. Wu et al. [41] in an in-vitro study of nerve cells, showed that high glucose concentrations could reduce or prevent the unfavorable effects of exposure to tumor necrosis factor alpha which include metabolic dysfunction, nuclear factor kappa beta activation, and inflammatory cytokine upregulation.

The current literature suggests the limited usefulness of clinical features or diagnostic studies to predict a favorable outcome after revision surgery, whether for incomplete release of FR or for recurrence CTS. Patients with an incomplete release of FR would logically experience similar outcomes as primary surgery, however, incomplete release at the primary surgery does not correlate well with outcome after revision surgery [10,42]. In part, this may be explained by unpredictable outcomes in severe patients with circumferential-fibrosis-related, vascular insufficiency, or traction injury to the MN. Patients with recurrent CTS will typically experience a more favorable outcome following revision surgery [11,43]. Patients having short or transverse incisions, nocturnal symptoms, symptoms exacerbated by activity, or a positive Phalen’s sign were shown to have favorable outcomes after revision surgery [30]. Beck et al. [10] suggest that gender, age, hand dominance, history of smoking or trauma, numbness/tingling, weakness, and pain all fail to be significant outcome predictors for revision surgery. Our data suggest that D5HD is particularly effective in recurrent CTS. Perineurial adhesions are a common contributing factor in recurrent CTS, and hydrodissection can extricate the entrapped MN from the surrounding adhesive/compressive tissue to further increase the blood flow and ameliorate nerve compression injury in recurrent CTS versus persistent CTS [19,33,44]. Moreover, our short-axis approach to simultaneously dissect above and below the MN, followed by a long-axis approach to hydrodissect proximally to distally through the carpal tunnel, may be particularly effective for releasing adhesive MN [20].

In 2021, we reported outcomes retrospectively in 185 patients receiving D5HD (a mean of 2.2 injection sessions using 10 mL 5% dextrose) over a mean of 15.8 months (range 1 to 3 years) post-injection follow-up. Data showed that 88.6% of patients reported symptom relief > 50%, without any adverse effects [17]. Only 1% (2/185) of the patients ultimately received surgery. That study included only 15/185 (8%) that had persistent or recurrent symptoms after surgery. Patients with mild, moderate and severe grades, respectively, required a mean of 1.7, 2.4, and 2.6 injections to achieve an effective outcome. In this data collection, exclusively from those that failed surgery, more treatment sessions (a mean of 3.1) were required to reach an effective outcome. We defined an effective outcome as symptom relief ≥ 50% compared to pre-injection, based on our previous studies [17,24]. This criterion for an effective outcome is higher than the minimal clinical important difference (MCID) of VAS which is a minimum decrease of 2 points [45] or a 25% reduction pain intensity [46]. No patient reported worsening after D5HD. Although 14 of 36 patients (38.9%) reported poor outcomes, six out of fourteen patients reported improvements in the MCID level (25% pain reduction). It would be premature to claim that D5HD is effective for patients with failure of CTS surgery, based on a retrospective study alone via subjective data obtained from phone interviews, and nearly 40% of patients did not achieve the criteria of an effective outcome. Hence, further studies with larger sample sizes are needed.

Increasing evidence has suggested an important role of neurogenic inflammation in the pathophysiology of neuropathic pain [47]. During the inflammatory response to nerve injuries, various inflammatory mediators, such as cytokines and chemokines are released by damaged cells and immune cells in the microenvironment of the lesion, which in turn induce painful neuropathy [47]. An increase in the levels of inflammatory cytokines and adhesion molecules has been documented in patients on hemodialysis that develop CTS [48]. Enhanced expression of growth factors, such as transforming growth factor (TGF-β), vascular endothelial growth factor (VEGF), and interleukins are found in CTS patients in the presence or absence of diabetes [49]. In contrast, a noninflammatory ischemia-reperfusion mechanism has been implicated in the progressive edema and fibrosis of SSCT, with adhesions and impaired gliding function of the MN [50,51]. Throughout the long-term therapeutic effect in our study, we hypothesize that mechanical hydrodissection played a predominant role in initial symptom relief, followed by a possible pharmacological effect of glucose to reduce the recurrence and promote neuroregeneration, enhancing long-term efficacy.

The study had several limitations. Our sample size is still relatively small, and we cannot entirely exclude the possibility of spontaneous remission, although this is unlikely to be a significant factor given the lengthy period between D5HD and surgery. The retrospective nature of this study, with its accompanying lack of standardized CTS outcome tool use, clinical data, such as physical examinations, follow-up ultrasonographic, or electrophysiological data at different time points, are limiting factors. However, significant differences in objective measures do not consistently predict clinically important outcomes [52]. Specifically, studies including electrophysiological or ultrasonographic outcome measures suggest either no, or only a limited, correlation with a patient’s clinical status or satisfaction after CTS surgery [53,54,55,56]. In contrast, previous literature shows a correlation between satisfaction and clinical improvement [57]. Hence, we believe that the lack of objective measurements in our study would not limit the value of clinical results. A recall bias in a retrospective study with a long follow-up duration is expected. It is difficult for patients to recall an initial absolute VAS score and then compare it with the current VAS score. A perceived percent improvement within stated ranges may be a better measurement than an estimate of exact percentage improvement [58], and is an appropriate way to ask the patient how much the symptoms improved compared with the pre-injection symptoms [17,24].

## 5. Conclusions

D5HD may result in clinically important and durable benefits in those experiencing persistent and recurrent CTS after surgery in this retrospective study of consecutive patients. Other prospective data gathering including more clinical, ultrasonographic, and potentially elastography data, is required to verify its therapeutic efficacy.

## Figures and Tables

**Table 1:** Demographic information of all patients and different outcomes.

	All Patients (*n* = 36)	Effective Outcome (*n* = 22)	Poor Outcome (*n* = 14)	^a^*p* Value
Gender, *n* (%)				0.217
Female	28 (77.8)	19 (86.4)	9 (64.3)	
Male	8 (22.2)	3 (13.6)	5 (35.7)	
Age (year) ± SE (range)	59.2 ± 1.6 (34–77)	57.8 ± 1.9 (34–67)	61.4 ± 2.7 (39–77)	0.267
BH (cm) ± SE (range)	157.6 ± 1.0 (148–174)	158.3 ± 1.5 (148–174)	156.5 ± 1.3 (150–165)	0.810
BW (kg) ± SE (range)	62.6 ± 1.9 (45–93)	63.4 ± 2.8 (46–93)	61.4 ± 2.4 (45–75)	0.936
DM (%)	10 (27.8)	6 (27.3)	4 (28.6)	0.932
Hypertension (%)	8 (22.2)	6 (27.3)	2 (14.3)	0.441
Lesion site, *n* (%)				0.879
Left	16 (44.4)	10 (45.5)	6 (42.9)	
Right	20 (55.6)	12 (54.5)	8 (57.1)	
Duration (month) ± SE (range)	15.1 ± 2.4 (1–48)	15.1 ± 3.1 (2–40)	15.1 ± 4.1 (1–48)	0.689
Classification (%)				0.175
Persistent	23 (63.9)	12 (54.5)	11 (78.6)	
Recurrent	13 (36.1)	10 (45.5)	3 (21.4)	
CSA (mm^2^) (SE)	14.1 ± 0.8	14.3 ± 1.2	13.6 ± 0.9	0.835
No. of injection (SE)	3.1 ± 0.3	3.1 ± 0.5	3.2 ± 0.5	0.597
Other treatment, (%)	11 (30.6)	5 (22.7)	6 (42.9)	0.671
Follow-up period (month), (SE) (range)	33.0 ± 2.8 (6–67)	35.9 ± 3.6 (7–67)	28.5 ± 4.4 (6–55)	0.160

BH = Body height; BW = Body weight; DM = Diabetes mellitus; CSA = Cross-sectional area; SE = standard error. ^a^ Mann–Whitney *U* Test, Fisher’s exact test.

**Table 2:** Demographic information of persistent and recurrent patients.

	Persistent (*n* = 23)	Recurrence (*n* = 13)	^a^*p* Value
Gender, *n* (%)			0.682
Female	17 (73.9)	11 (84.6)	
Male	6 (26.1)	2 (15.4)	
Age (year) ± SE (range)	57.6 ± 2.3 (34–77)	61.9 ± 1.2 (55–69)	0.281
BH (cm) ± SE (range)	158.6 ± 0.9 (150–170)	155.9 ± 2.3 (148–174)	0.006
BW (kg) ± SE (range)	62.7 ± 1.6 (46–75)	62.5 ± 4.7 (45–93)	0.361
DM (%)	6 (26.1)	4 (30.8)	0.763
Hypertension (%)	4 (17.4)	4 (30.8)	0.422
Lesion site, *n* (%)			0.731
Left	11 (47.8)	5 (38.5)	
Right	12 (52.2)	8 (61.5)	
Duration (month) ± SE (range)	17.0 ± 3.3 (1–48)	11.7 ± 3.2 (2–36)	0.580
CSA (mm^2^) (SE)	13.9 ± 0.8	14.3 ± 1.9	0.474
No. of injection (SE)	3.2 ± 0.5	3.0 ± 0.4	0.845
Outcome			0.165
Effective	12 (52.2)	10 (76.9)	
Poor	11 (47.8)	3 (23.1)	
Other treatment, (%)	6 (26.1)	5 (38.5)	0.475
Follow-up period, (months) (SE) (range)	30.7 ± 3.1 (6–67)	37.2 ± 5.5 (7–62)	0.281

BH = Body height; BW = Body weight; DM = Diabetes mellitus; CSA = Cross-sectional area; SE = standard error. ^a^ Mann–Whitney *U* Test, Fisher’s exact test.

**Table 3:** Demographic information of all patients with different follow-up periods.

	<2 Years (*n* = 11)	2–4 Years (*n* = 18)	>4 Years (*n* = 7)	^a^*p* Value
Gender, *n* (%)				0.182
Female	7 (63.6)	14 (77.8)	7 (100)	
Male	4 (36.4)	4 (22.2)	0 (0)	
Age (year) ± SE (range)	59.5 ± 2.1 (48–77)	57.8 ± 2.8 (34–75)	62.3 ± 1.3 (56–66)	0.454
BH (cm) ± SE (range)	157.1 ± 1.5 (148–165)	159.4 ± 1.6 (150–174)	153.6 ± 1.0 (150–157)	0.810
BW (kg) ± SE (range)	63.0 ± 2.8 (45–75)	64.7 ± 3.1 (46–93)	56.6 ± 4.0 (45–76)	0.936
DM (%)	1 (9.1)	8 (44.4)	1 (14.3)	0.104
Hypertension (%)	2 (18.2)	6 (33.3)	0 (0)	0.216
Lesion site, *n* (%)				0.903
Left	4 (36.4)	9 (50.0)	3 (42.9)	
Right	7 (63.6)	9 (50.0)	4 (57.1)	
Duration (month) ± SE (range)	10.4 ± 4.3 (1–48)	12.7 ± 2.9 (2–38)	28.6 ± 5.6 (12–48)	0.016
Classification (%)				0.113
Persistent	7 (63.6)	14 (77.8)	2 (28.6)	
Recurrence	4 (36.4)	4 (22.2)	5 (71.4)	
CSA (mm^2^) (SE)	14.0 ± 1.1	15.1 ± 1.4	11.6 ± 1.0	0.414
No. of injection (SE)	3.7 ± 0.5	3.1 ± 0.5	2.3 ± 0.6	0.206
Outcome				0.077
Effective	4 (36.4)	14 (77.8)	4 (57.1)	
Poor	7 (63.6)	4 (22.2)	3 (42.9)	
Other treatment, (%)	2 (18.2)	7 (38.9)	2 (28.6)	0.640
Follow-up period (months) (SE) (range)	14.6 ± 2.1 (6–23)	35.1 ± 2.0 (25–47)	56.7 ± 2.6 (50–67)	<0.001

BH = Body height; BW = Body weight; DM = Diabetes mellitus; CSA = Cross-sectional area; SE = standard error. ^a^ Kruskal–Wallis test, Fisher’s Exact Test.

## Data Availability

The data presented in this study are available on request from the corresponding author.
